# Correction: Epidemiological dynamics of Ebola outbreaks

**DOI:** 10.7554/eLife.05419

**Published:** 2014-11-07

**Authors:** Thomas House

House T. 2014. Epidemiological dynamics of Ebola outbreaks. *eLife* **3**:e03908. doi: 10.7554/eLife.03908. Published 12 September 2014

In the published article text, a term was omitted from Equation 2. This equation read:

$$\text{Pr}\left[Z=z\text{|}A=a,q\right]=\left(\begin{array}{c} 2z+a-1\\ z \end{array}\right)q^{z+\text{a}}{\left(1-q\right)}^{z}$$

It should have read:

$$\text{Pr}\left[Z=z\text{|}A=a,q\right]=\frac{a}{a+z}\left(\begin{array}{c} 2z+a-1\\ z \end{array}\right)q^{z+\text{a}}{\left(1-q\right)}^{z}$$

The article has now been corrected.

